# Regulation of phosphate starvation-specific responses in *Escherichia coli*

**DOI:** 10.1099/mic.0.001312

**Published:** 2023-03-27

**Authors:** Patrice L. Moreau

**Affiliations:** ^1^​ Laboratoire Chimie Bactérienne, LCB-UMR 7283, Institut Microbiologie Méditerranée, CNRS/Université Aix-Marseille, Marseille, France

**Keywords:** PtsN, Kdp, LexA, SoxR, CpxR, RpoE

## Abstract

Toxic agents added into the medium of rapidly growing *Escherichia coli* induce specific stress responses through the activation of specialized transcription factors. Each transcription factor and downstream regulon (e.g. SoxR) are linked to a unique stress (e.g. superoxide stress). Cells starved of phosphate induce several specific stress regulons during the transition to stationary phase when the growth rate is steadily declining. Whereas the regulatory cascades leading to the expression of specific stress regulons are well known in rapidly growing cells stressed by toxic products, they are poorly understood in cells starved of phosphate. The intent of this review is to both describe the unique mechanisms of activation of specialized transcription factors and discuss signalling cascades leading to the induction of specific stress regulons in phosphate-starved cells. Finally, I discuss unique defence mechanisms that could be induced in cells starved of ammonium and glucose.

## Introduction

In nature, environmental conditions can change frequently as a result of fluctuations in growth parameters − e.g. carbon source [1], phosphate source [2], osmotic pressure and potassium concentration [3], iron availability [4], pH [5], temperature [6] and oxygen level [7] − and production of toxic products by competing organisms [8–11].

Changes in environmental conditions can directly (e.g. addition of toxic products) [12, 13] and indirectly (e.g. starvation of carbon and phosphate) [14] generate potentially lethal damage. Bacteria evolved different mechanisms to survive whether they are growing or non-growing (i.e. in stationary phase).

In rapidly growing cells exposed to toxic products, specific stress responses are induced through the activation of specialized transcription factors that recruit the vegetative RNA polymerase (RNAP)-σ^70^ holoenzyme to promoters of genes required for stress survival. Each transcription factor and downstream regulon (e.g. SoxR, CpxR and LexA) are specific to a unique type of stress (e.g. superoxide stress, envelope stress and DNA replication stress) [6, 9, 15]. In addition, high temperature can induce the RpoE and RpoH regulons through activation of the specialized sigma factors σ^E^ and σ^H^, respectively [6].

In cells starved of glucose (Glc) and inorganic phosphate (Pi) that enter into stationary phase, a single general stress response is induced through the accumulation of the sigma factor σ^S^ (RpoS). The RNAPσ^s^ holoenzyme induces the RpoS regulon that include many genes (e.g. *sodC*, *katE*, *dps*, *pdhR*, *poxB* and *gadAB*) that help to protect non-growing cells against a variety of stresses (e.g. oxidative stress and acid stress) [16–19].

The distinction between specific stress responses induced by toxic products in growing cells and the general stress response induced in non-growing cells was blurred by the finding that specific stress regulons can be induced in cells that approach the stationary phase in the absence of added toxic products in the environment: CpxR is activated in cells that enter into stationary phase during incubation in rich medium supplemented with glucose [20, 21]; RpoE is activated in cells starved of amino acids and Pi [22, 23]; and SoxR, OxyR, CpxR, RpoE and LexA are activated under Pi-starvation conditions [14, 24–26]. The regulatory cascades leading to the activation of specific stress regulons under growth-limiting conditions remain poorly understood.

In this review, the focus is on *Escherichia coli* K-12 starved of Pi. I review the unique mechanisms of activation of the SoxR-, CpxR-, RpoE- and LexA-specific transcription factors, and I attempt to answer the following questions: What are the signalling pathways leading to induction of the specific stress regulons? What are the roles of induced genes? Why are specific stress regulons induced rather than the general stress response? Finally, I discuss unique regulatory cascades that could be induced in cells starved of ammonium and glucose.

## Induction of oxidative stress regulons in Pi-starved cells

*E. coli* evolved two complementary oxidative stress regulons, SoxRS (e.g. *sodA*, *zwf*, *acnA*, *acrAB*, *micF* and *poxB*) and OxyR (e.g. *ahpCF, katG, dps, trxC, grxA* and *sufA-E*), whose aim is to protect cytoplasmic components from oxidative damage [15].

In growing cells, the SoxRS-specific stress response is induced by redox-cycling compounds added to the environment (e.g. paraquat and plumbagin) [10, 27], which help transfer one electron from SoxR − i.e. oxidation of SoxR − to molecular oxygen − i.e. reduction of O_2_ to superoxide (O_2_^•−^). Superoxide is a reactive oxygen species (ROS) that damages metabolic enzymes containing unique iron-sulphur cluster (FeS) [15]. The oxidized form of SoxR (SoxR^ox^) induces the synthesis of SoxS, which induces genes (e.g. *sodA* and *zwf*) that help combat oxidative stress. SodA detoxifies superoxide (O_2_^•−^) through its dismutation into molecular oxygen (O_2_) and hydrogen peroxide (H_2_O_2_). Zwf (NADP-dependent glucose-6-phosphate dehydrogenase), the first enzyme of the pentose phosphate pathway (PPP), produces NADPH that is required for the activity of reducing systems (e.g. Trx/Grx) including Rsx, which reduces SoxR^ox^ [27].

In Pi-starved cells, induction of the SoxRS system is dependent upon the expression of the Rsx-reducing system, which prevents over-oxidation and eventually destruction of the [2Fe-2S] cluster in SoxR [26]. Therefore, contrarily to the hypothesis that SoxR could be oxidized only through interaction with redox-cycling compounds added to the growth medium [10], it is likely that SoxR could be activated as a result of the endogenous production of ROS in cells starved of Pi [26].

### What enzymes could generate ROS in Pi-starved cells?

**Fig. 1.**
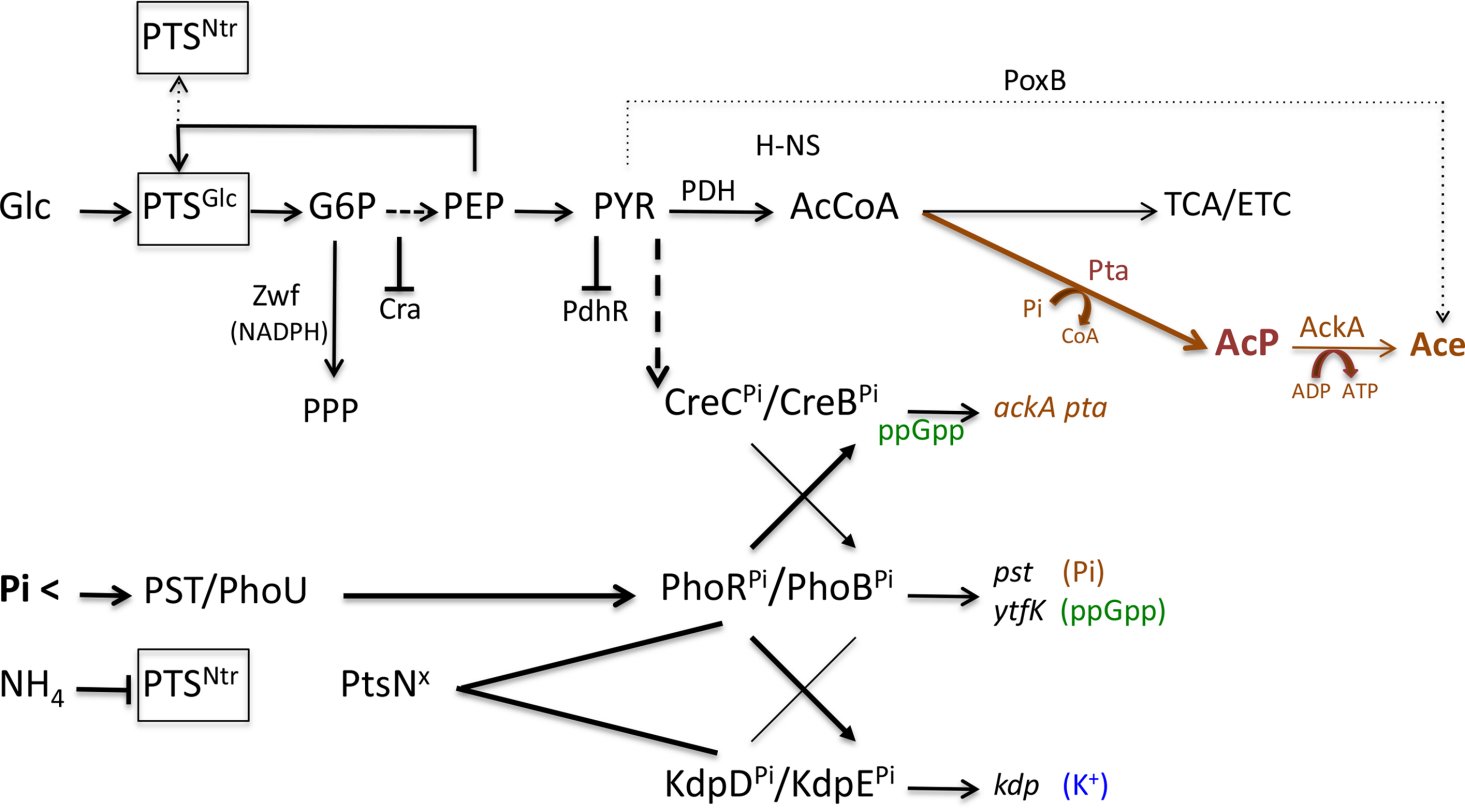
Changes in metabolic pathways in Pi-starved cells during the transition to the stationary phase. PTS^Glc^ transports and phosphorylates Glc. Zwf (NADP-dependent glucose-6-phosphate dehydrogenase) partly directs the flow of G6P into PPP. Metabolites of central carbon metabolism inhibit Cra and PdhR DNA-binding activities, and activate the CreC/CreB TCS. The PYR-AcCoA flux is largely redirected from the PDH-TCA-ETC pathway (aerobic respiration) towards the PDH-Pta-AckA pathway (aerobic fermentation; in brown). Under shortage of Pi (Pi <), the PhoR/PhoB TCS stimulates primarily the scavenging of Pi (in brown), as well as the synthesis of ppGpp (in green). Under excess ammonium conditions, the nonphosphorylated form of PtsN (PtsN^x^) interacts with and favours the active phosphorylated forms of PhoR^Pi^ and KdpD^Pi^. Cross talks between PhoR/PhoB, KdpD/KdpE and CreC/CreB TCSs induce the *kdpABC* and *ackA pta* operons. Abbreviations: AcCoA, acetyl-coenzyme A; Ace, acetate/acetic acid; AcP, acetyl phosphate; ETC, electron-transfer chain; Glc, glucose; G6P, glucose-6-phosphate; PDH, pyruvate dehydrogenase complex; PPP, pentose phosphate pathway; PEP, phospho*enol*pyruvate; Pi, inorganic phosphate; PTS, phosphotransferase *s*ystem; PST, phosphate specific transporter; PYR, pyruvate; TCA, tricarboxylic acid cycle.

Three lines of evidence suggest that redox enzymes could generate ROS. First, between days 3 and 6 of incubation under Pi-starvation conditions, the accumulation of oxidation products (i.e. thiobarbituric acid reactive substances, TBARS) is prevented through the induction of *poxB* in a strictly RpoS-dependent manner [28]. PoxB (pyruvate:quinone oxidoreductase) directly oxidizes pyruvate (PYR) into acetate (Ace), which prevents PYR flux through the NAD-dependent pyruvate dehydrogenase complex (PDH) [7]. PDH converts PYR into acetyl-coenzyme A (AcCoA), which can enter into the tricarboxylic acid cycle (TCA). PDH and TCA activities are linked to the activity of aerobic electron-transfer chain (ETC), which reduces NADH [7] (Fig. 1). Second, a peak in the production of NAD^+^ is observed after 3 h in stationary phase under Pi-starvation conditions [14]. NAD^+^ biosynthesis, which liberates Pi (NadA, NadC, NadD and NadE activities) [29], could transiently support the activity of metabolic enzymes that require Pi and/or NAD^+^ such as glyceraldehyde-3-phosphate dehydrogenase in glycolysis, PDH (AceEF-Lpd), and α-ketoglutarate dehydrogenase (KGDH, SucAB-Lpd) in TCA [30, 31]. Third, solvent exposed FADH_2_ in NadB (in NAD^+^ biosynthetic pathway), SdhABCD (succinate:quinone oxidoreductase in TCA) and Ndh (NADH:quinone reductase II in ETC) can accidentally transfer electrons to molecular oxygen, which generates superoxide, hydrogen peroxide and hydroxyl radicals [10, 15, 29, 32]. It is therefore tempting to speculate that aerobic glucose metabolism through the PDH-TCA-ETC pathway could directly (Sdh and Ndh) and indirectly (NadB) generate ROS in Pi-starved cells before entry into stationary phase and induction of the PoxB bypass.

Hydrogen peroxide is actually produced to potentially toxic levels in Pi-starved cells. Survival of Pi-starved cells that approach the stationary phase is primarily dependent upon the activity of the AhpCF peroxidase [14], which can reduce low levels H_2_O_2_ [10]. Whereas low levels hydrogen peroxide can oxidize (i.e. activate) OxyR, even high levels of superoxide cannot directly oxidize SoxR [10, 15, 33].

### How is SoxR oxidized in Pi-starved cells?

Hydrogen peroxide, a generally weak oxidant, can generate a very toxic product, the hydroxyl radical. Hydrogen peroxide can oxidize free ferrous iron (Fe**^2+^**) to ferric iron (Fe**^3+^**), which reduces H_2_O_2_ (HO**••**OH +1 e^−^) to hydroxide ion (HO**••**) and hydroxyl radical (HO**•**) (Fenton reaction) [15]. The Fenton reaction is more efficient when cells are exposed to low doses of H_2_O_2_ for a long time − as in Pi-starved cells − rather than to high doses for a short time [33].

**Fig. 2.**
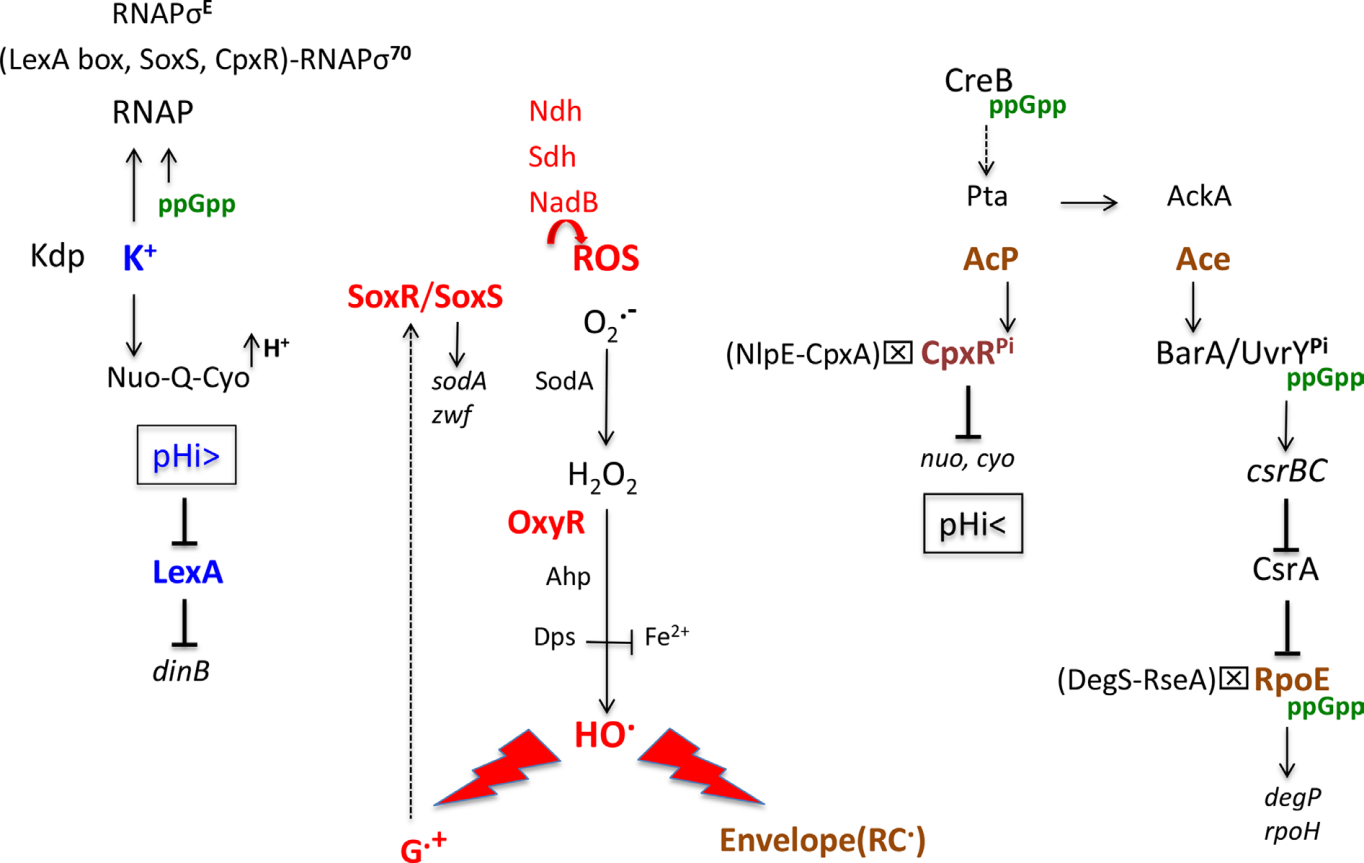
Induction of specific stress regulons in Pi-starved cells during the transition to the stationary phase. The flavoenzymes Ndh, SdhCDAB and NadB generate ROS (bend arrow in red). Low levels hydrogen peroxide (H_2_O_2_) activate OxyR. Hydroxyl radicals (HO•) damage membrane proteins (RC•) and guanine residues in DNA. G^•+^ oxidizes SoxR. Activation of the Kdp system (K**^+^**) (in blue) triggers alkalinization of the cytoplasm, which decreases the binding affinity of the LexA repressor to LexA box. The Pta-AckA pathway produces AcP and Ace (in brown), which activate the envelope stress regulons CpxR and RpoE, respectively. The alarmone ppGpp (in green) helps to induce specific stress regulons. The cross in a box indicates that activation of CpxR and RpoE occurs independently of the NlpE-CpxA and DegS-RseA pathways, respectively. Abbreviations: Ace, acetate/acetic acid; AcP, acetyl phosphate; pHi, internal pH; RNAP, RNA polymerase; ROS, reactive oxygen species.

The hydroxyl radical readily abstracts one electron (hydrogen atom) from proteins and nucleic acids (HO• + RCH → HO**••**H + RC•), which can eventually result in aggregation (RC••CR) and fragmentation of oxidized molecules [12]. The primary target of hydroxyl radical is directly and indirectly guanine residue in DNA (HO• + G → HO**••**H + G^•+^) [13]. Activation of SoxR could therefore occur through DNA charge transport [13, 34] of one electron from SoxR, bound to the promoter of *soxS,* to a close oxidized guanine, which could reduce G^•+^ and oxidize SoxR (G^•+^ + SoxR^red^→ G + SoxR^ox^) [26, 28] (Fig. 2). SoxR^ox^ should be rapidly reduced by the NADPH-dependent Rsx system to prevent the degradation of its [2Fe-2S] cluster, thereby entering into a new redox cycle [7, 26, 27]. If not reduced by DNA charge transport, G^•+^ develops to miscoding lesion (8-oxoGua) and noncoding lesion − fragmentation of guanine into FapyGua blocks DNA replication [13].

## Induction of envelope stress regulons in Pi-starved cells

*E. coli* evolved two complementary envelope stress responses, CpxR and RpoE, whose aim is primarily to prevent the integration of misfolded proteins into inner and outer membranes [6]. The CpxR and RpoE regulons are induced through unique mechanisms in Pi-starved cells [25].

### Activation of the CpxR regulon

In growing cells, the CpxA/CpxR two-component system (TCS) is activated under environmental conditions that damage envelope proteins (e.g. alkaline pH and addition of gentamicin, an aminoglycoside antibiotics) [6, 25]. ATP-dependent auto-phosphorylation of CpxA can be triggered following faulty interaction between CpxA and the lipoprotein NlpE, when notably the biogenesis and translocation of NlpE into the outer membrane is hampered [35]. Phospho-CpxR (CpxR^Pi^) induces primarily genes required for the elimination of abnormal proteins in the periplasm. CpxR^Pi^ can also repress the transcription of genes implicated in ETC biogenesis, namely *nuo* and *cyo* encoding NADH:quinone reductase I and cytochrome-quinol oxidase *bo*_3_, respectively [36]. The composition of ETCs adapts to growth conditions [7]. ETCs are composed of NADH:quinone reductases (Ndh and Nuo), quinones (Q) and cytochrome-quinol oxidases (Cyo and Cyd), which consume and excrete various amounts of protons. The repression of *nuo* and *cyo* by CpxR decreases the excretion of protons (from a maximum of 8 H^+^/2 e^-^ with the Nuo-Q-Cyo combination down to 2 H^+^/2 e^-^ with the Ndh-Q-Cyd combination), which decreases the internal pH (pHi). The repression of *nuo* and *cyo* can explain that induction of the CpxR envelope stress response by the antibiotic gentamicin decreases the pHi [11]. Globally, the inhibition of protons excretion can decrease both the ΔpH and the Δψ (transmembrane electrical potential), which compose the proton motive force (PMF) − pHi is alkaline and the inner surface of the cytoplasmic membrane is negative in cells growing in minimal medium at pH 7 [5, 7, 37]. Induction of the CpxR response can therefore mitigate an increase in the pHi under alkaline growth conditions, and prevent Δψ-dependent entry of gentamicin into the cytoplasm [25, 36].

In Pi-starved cells, induction of the CpxR regulon is independent of the CpxA kinase activity but is dependent upon PhoB activity [25]. There is evidence that CpxR can be phosphorylated directly by acetyl phosphate (AcP) in cells grown in amino-acid-rich medium supplemented with glucose [20, 21]. At the approach of the stationary phase, when preferred amino acids are consumed, the switch to glucose metabolism generates AcP, an efficient phosphoryl group donor [38, 39].

During rapid growth on glucose, PYR and AcCoA flux is mainly directed towards the fermentative phosphate acetyltransferase-acetate kinase (Pta-AckA) pathway, so-called overflow metabolism, rather than toward the aerobic TCA-ETC pathway [40]. Pta converts AcCoA and Pi into CoASH and AcP; AckA converts AcP and ADP into Ace and ATP [20, 38, 40]. The Pta-AckA pathway can therefore provide energy rapidly and at low proteome cost during growth on glucose [41]. A major regulator of overflow metabolism is the CreC/CreB TCS (*C*arbon source *re*sponsive), which somehow senses the accumulation of PYR and responds with induction of the *ackA-pta* operon [42] (Fig. 1).

AcP transiently accumulated in cells grown in rich medium supplemented with glucose can therefore phosphorylate CpxR (CpxR^Pi^) [20, 21, 38]. The same process could occur in Pi-starved cells that approach the stationary phase when the incubation medium contains excess glucose [14]. The Pta-AckA pathway could be the primary source of energy, and AcP could be partly used to phosphorylate CpxR (Fig. 2). Pi-starved cells accumulate high levels of AcP when they enter into stationary phase [43].

What is the role of the PhoR/PhoB response in CpxR activation? When the concentration of Pi decreases below 4 µM in the medium, changes in the structure of the primary *p*hosphate *s*pecific *t*ransporter PstSCAB (PST) activate the PhoR/PhoB TCS [44]. The PhoR/PhoB system could stimulate the transcription of the *ackA-pta* operon and the production of AcP at three different levels (Fig. 1). First, PhoB^Pi^ induces the *pst* and *phn* operons, which help to scavenge the Pi required for AcP synthesis [2, 38, 45]. Second, PhoR^Pi^ can phosphorylate the non-cognate regulator CreB [42]. Third, PhoB^Pi^ induces *ytfK*, which can help CreB^Pi^ to transcribe the *ackA-pta* operon [46, 47]. YtfK interacts with SpoT, which increases the production of the nucleotide second messenger Guanosine tetraphosphate (ppGpp) [46]: ppGpp stimulates the transcription of the *ackA-pta* operon [47]. Generally, the production of ppGpp depends upon changes in RelA and SpoT activities in response to low translation rate when the growth rate decreases [48]. The alarmone ppGpp primarily binds to RNAP, which adjusts metabolism to poor nutrient availability and stress conditions [49]. Therefore, induction of the PhoR/PhoB TCS may be required to help activate CreB^Pi^, induce the *ackA-pta* operon, and accumulate AcP (Fig. 1), which phosphorylates CpxR (Fig. 2).

The CpxA/CpxR and BaeS/BaeR envelope stress responses are both induced in the presence of ethanol [6, 50, 51]. The role of the BaeSR-MdtABC efflux pump system, which is specific to natural products [50], was not tested in Pi-starved cells. There is no evidence of direct phosphorylation of BaeR by AcP.

### Activation of the RpoE regulon

In growing cells, the RpoE envelope stress response is primarily induced by high temperature [6]. The RpoE response results from activation of the sigma factor σ^E^ (σ^24^). RpoE (σ^E^) activity depends on its release from the anti-sigma factor RseA. The protease DegS, activated by the presence of abnormal proteins in the periplasm, degrades RseA, which frees RpoE. The RpoE regulon (e.g. *lpxD, degP* and *clpX lon*) encodes proteins ensuring the integrity of the outer membrane through the synthesis of lipopolysaccharide and proteolysis of misfolded outer-membrane porins [52]. Moreover, the RpoE response indirectly helps to eliminate abnormal proteins in the cytoplasm through the induction of *rpoH*, which encodes the heat-shock protein regulator RpoH (σ^32^). Heat-shock proteins include ATP-dependent molecular chaperones and proteases (e.g. the ClpXP degradation complex and Lon) that refold and eventually degrade proteins damaged by high temperature, extreme pH and oxidative stress [53].

In cells starved of Pi, activation of the RNAPσ^E^ holoenzyme is independent of RseA and PhoB [22, 25]. There is evidence that the alarmone ppGpp could play a key role in increasing RpoE levels through the Csr (*C*arbon *s*torage *r*egulation) system. High levels of ppGpp increase the RNAPσ^70^-dependent transcription of *csrB* and *csrC*; the CsrB and CsrC small RNAs bind to and inactivate the activity of CrsA, which inhibits the translation of the *rpoE* mRNA [54]. In addition to increasing RpoE levels, ppGpp can increase the activity of the RNAPσ^E^ holoenzyme [22, 23]. However, ppGpp levels cannot totally explain the activation of the RpoE regulon in Pi-starved cells, which suggests the presence of another factor [22]. Besides ppGpp, the BarA/UvrY TCS can also induce the *csrB and csrC* genes [55]. The BarA/UvrY system senses and responds to high levels of Ace that can be produced by the Pta-AckA pathway in Pi-starved cells [28, 56]. Before activation of the BarA/UvrY TCS by Ace, the response regulator UvrY could be directly phosphorylated by AcP [55], which could induce the CsrBC/A system before the accumulation of toxic levels of acetic acid. Therefore, both UvrY^Pi^ and ppGpp might be required to increase RpoE levels and RNAPσ^E^ activity independently of RseA activity in Pi-starved cells (Fig. 2).

How is the RpoE regulon induced in *phoBR* mutants? The production of Ace results from the activity at first of the Pta-AckA pathway (when cells approach the stationary phase) and later on of the PoxB pathway (when cells enter into stationary phase). Each pathway contributes equally to Ace final concentration in the presence of excess glucose [28]. If Pta activity (and AcP levels) were low in *phoBR* mutants − as suggested by inhibition of the CpxR activity − induction of *poxB* by the RNAPσ^S^ holoenzyme could occur prematurely as a result of a rapid drop in growth rate and entry into stationary phase. PoxB-dependent production of Ace could induce the BarA/UvrY TCS, which might account for induction of the RpoE regulon in *phoBR* mutants starved of Pi [22, 25]. If high levels of ppGpp were required, PhoB-dependent induction of *ytfK* should play a minor role. Loss of YtfK-dependent activation of SpoT could be compensated by activation of RelA (and SpoT) in response to a rapid drop in growth rate [48, 49].

## Induction of the LexA regulon in Pi-starved cells

In rapidly growing cells, the LexA response is induced when DNA bulky lesions − produced for instance by the antibiotic mitomycin C − stall the replicative DNA polymerase Pol III. Reinitiating replication beyond lesions generates single-stranded DNA (ssDNA) gaps [57]. Induction of the LexA regulon (e.g. *lexA*, *recA*, *dinB*, *polB* and *umuDC*) results from the (auto)cleavage of the LexA repressor when free LexA molecules interact with short RecA-ATP filaments built on ssDNA organized by the ssDNA-binding protein (SSB) [58–61]. The LexA response primarily helps to tolerate DNA damage through the activity of error-prone DNA polymerases: Pol IV (DinB) − possibly in a complex with RecA and native UmuD − can replicate damaged DNA, whereas Pol V (UmuD'C) can fill ssDNA gaps [62–64]. Gap filling can eventually occur through homologous recombination initiated by long RecA-ATP-ssDNA filaments formed through interaction between SSB and RecF [65–67].

The LexA regulon can be induced when cells approach the stationary phase in the absence of added genotoxic products in the medium. This occurs when cells are grown in rich LB medium − pH sharply increases at the approach of the stationary phase as a result of amino-acid degradation − and when cells are starved of Pi but not when they are starved of ammonium or glucose [24]. Contrary to the current dogma that induction of the LexA regulon requires LexA cleavage [9, 68–70], induction of the LexA regulon in starved cells is independent of LexA cleavage but is dependent upon cytoplasm alkalinization [39]. An exposed histidine residue in LexA, which contacts the DNA phosphodiester backbone near the LexA box, could mainly exist in deprotonated form under alkaline conditions [69, 71, 72], which could reduce the affinity of the LexA repressor for LexA-controlled promoters [39].

Therefore, the LexA regulon can be markedly induced in Pi-starved cells in the absence of the ssDNA signalling system through an increase in the pHi, which decreases the binding affinity of LexA to LexA box [39]. In contrast, a decrease in the pHi triggered by the addition of antibiotics could increase the binding affinity of RecA to ssDNA, which stimulates the cleavage of LexA [11, 73].

### How could the pHi increase in cells starved of Pi, whereas ongoing glucose metabolism eventually causes the accumulation of acetic acid?

Ace excreted into the medium decreases the pH of the medium during prolonged incubation of Pi-starved cells. At moderate acidic pH, acetic acid present in the medium diffuses across the membrane and dissociates into acetate (AcO^-^) and protons (H^+^) inside the cell [56]. However, the pHi could increase at the approach of the stationary phase as a result of a perturbation in the K^+^/H^+^ ion balance.

Potassium, which is normally transported by the Trk system (K^+^:H^+^ symport), is the most abundant intracellular cation. Potassium helps to control key physiological parameters such as osmotic pressure, ionic strength and pHi. When the concentration of potassium falls into the micromolar range in the medium, activation of the KdpD/KdpE TCS induces the *kdpFABC* operon, which encodes the Kdp high-affinity ATP-dependent K^+^ pump. Upon osmotic upshift, potassium uptake through the Trk and Kdp systems increases dramatically, which triggers the extrusion of protons and a transient alkalinization of the cytoplasm [3].

When Pi-starved cells approach the stationary phase, the *kdp* operon is induced whereas potassium is in excess in the medium [26]. Moreover, constitutive induction of the Kdp system in *kdpD^c^* mutants [26] enhances the level of expression of the LexA regulon under Pi-starvation conditions (PLM unpublished results), which suggests that activation of the Kdp transport system, accumulation of K^+^, and extrusion of protons (i.e. alkalinization of the pHi) could account for the non-canonical induction of the LexA regulon in Pi-starved cells (Fig. 1).

### How could induction of the *kdp* operon take place in Pi-starved cells?

Activation of the *kdp* operon could result from both a crosstalk between PhoR^Pi^ and KdpE [74], and formation of a PhoR^Pi^-KdpD^Pi^-PtsN ternary complex [75] (Fig. 1). PtsN is the last protein of the nitrogen-related phospho*enol*pyruvate phosphotransferase system (PTS^Ntr^). The components of the PTS^Ntr^ system are mainly in dephosphorylated form in cells incubated in a minimal medium containing excess ammonium [76]. In dephosphorylated form, PtsN^x^ interacts with and favours the active phosphorylated forms of both PhoR^Pi^ and KdpD^Pi^ [75].

### How could K^+^ accumulation alkalinize the cytoplasm of Pi-starved cells?

K^+^ ions specifically bind to and increase the activity of NADH:quinone reductase I (Nuo) [77], which can improve H^+^ excretion by ETC [3]. Therefore, induction of the *kdp* operon, K^+^ accumulation and stimulation of Nuo activity could account for alkalinization of the cytoplasm in Pi-starved cells (Fig. 2).

Besides its effect on the pHi, excess potassium could improve the expression of specific stress regulons through the redistribution of RNAP to promoters of induced genes. Exponentially growing cells exposed to a moderate osmotic up-shock accumulate rapidly potassium, which globally destabilizes RNAP-DNA complexes: RNAP can then re-associate with σ^70^ to promoters of osmo-regulated genes and eventually with σ^S^ [3, 78]. Similarly, potassium accumulated in Pi-starved cells could favour the re-association of RNAPσ^70^ to promoters that bind SoxR^ox^ and CpxR^Pi^, and to promoters made accessible following the release of the LexA repressor from LexA box (Fig. 2). Later, RNAP could re-associate with σ^E^ and σ^H^ when made available.

## Roles of specific stress regulons in Pi-starved cells

Flavoenzymes adventitiously produce ROS in Pi-starved cells. However, redirection of PYR metabolism from the PDH-TCA-ETC aerobic pathway towards the PDH-Pta-AckA fermentative pathway decreases the production of ROS by Sdh and Ndh in TCA and ETC, respectively. Because of activation of the NAD^+^ biosynthetic pathway (primarily to sustain NAD^+^-dependent PDH activity), NadB could be a primary source of ROS in Pi-starved cells [14, 28, 29, 32, 79] (Fig. 2). The superoxide dismutase SodA (SoxR regulon) could play a key role in protecting FeS-metabolic enzymes against superoxide anion radical (O_2_•^-^) [27, 80]. The peroxidase AhpCF (OxyR regulon) is required to reduce hydrogen peroxide (H_2_O_2_) and prevent the formation of toxic hydroxyl radicals (HO•) [14, 27]. Therefore, the primary role of LexA, CpxR and RpoE regulons could be to help SoxR and OxyR regulons to alleviate oxidative stress in Pi-starved cells.

In the LexA regulon, *dinB* encoding the Pol IV TLS DNA polymerase could play a key role. DinB helps to skip single-strand nicks generated through hydroxyl radical-mediated oxidative degradation of 2-deoxyribose moiety [13]. In the absence of DinB, nicks stall normal DNA replication and generate lethal double-strand breaks (DSB) [81]. Finally, induction of the CpxR and RpoE regulons could help to prevent the accumulation of oxidized proteins in the inner and outer membranes, respectively [6, 52].

Why did such mechanisms evolve since the RpoS response can efficiently protect Pi-starved cells against multiple stresses including oxidative stress? SoxR-, OxyR-, LexA- and CpxR-specific stress responses are induced through the activation of specialized transcription factors that recruit the vegetative RNAPσ^70^ holoenzyme to promoters of a limited number of genes. In sharp contrast, the RpoS-dependent general stress response dramatically remodels the transcriptome, which allows non-growing cells to fight against many stresses [19]. At first glance the general stress response could offer the best way to protect Pi-starved cells against oxidative stress through induction of *katE*, *sodC*, *gor*, *dps*, *dinB*, *poxB* and *pdhR*. However, the switch from RpoD (σ^70^)- to RpoS (σ^S^)-dependent transcription might be a risky investment. Exit from the stationary phase upon dilution into a rich medium requires a lag period to change metabolic pathways from a survival (RpoS-dependent) to a growth (RpoD-dependent) regimen [82, 83]. In a population of Pi-starved cells, cells in which the RpoS switch is delayed could have an advantage over cells that entered into stationary phase whether a Pi source is recovered and growth is possibly restarted [84].

The H-NS protein can delay the switch from RNAPσ^70^ to RNAPσ^S^ notably during incubation at low temperature [24, 28, 81]. High levels of H-NS and low Pta activity at low temperature [38, 43, 85] could account for the poorly defined finding that induction of the LexA regulon is dramatically increased in Pi-starved at low temperature [24]. High levels of H-NS can delay the switch from σ^70^ (RpoD) to σ^S^ (RpoS) (Fig. 1), and low Pta activity − resulting in low AcP levels and low CpxR activity − can delay the switch from alkaline (Kdp-dependent) to acidic pHi (CpxR-dependent) (Fig. 2), both conditions, which can favour the RNAPσ^70^-dependent expression of the LexA regulon in Pi-starved cells.

Taken together, these data support the idea that specific stress regulons could protect Pi-starved cells against oxidative stress before the RpoS switch. Specific stress regulons can protect notably DNA and proteins essential for metabolism and envelope structure. Such a protection might be critical whether Pi-starved find a new source of Pi and start growing rapidly. In other words, specific stress regulons can afford a transient protection against oxidative stress whether *E. coli* should adapt to frequent and rapid environmental changes in Pi concentrations, the so-called ‘feast and famine cycle’ [84], which occurs in the small intestine [45].

Because Pi starvation and ensuing oxidative stress are predictable events, *E. coli* evolved unique regulatory processes leading to induction of specific stress regulons before oxidative damage totally compromise protein synthesis [86, 87]. The signalling pathways leading to induction of LexA and CpxR/RpoE regulons share a common core constituted of PtsN^x^ and PhoR^Pi^, thereby allowing a co-ordinated induction (Fig. 1). However, the metabolic signals used − Pi limitation, excess ammonium and excess glucose − are characteristic of Pi-starvation conditions, which suggests that cells starved for other nutrients could behave differently. In fact, preliminary data suggest that the nature of stress systems and mechanisms of induction differ in Pi-, glucose- and ammonium-starved cells.

## Induction of the SoxRS regulon in ammonium-starved cells

In ammonium-starved cells, gene fusions used as reporters of stress regulons (Kdp, LexA and RpoE) are poorly expressed with the remarkable exception of the *soxS::lacZ* fusion. Induction of *soxS* is stronger in ammonium- than in Pi-starved cells. Moreover, expression of the *soxS::lacZ* fusion is more dependent upon the activity of the SoxR-reducing system Rsx in ammonium- than in Pi-starved cells, which indicates that oxidative stress is stronger in the former than in the latter [25, 26, PLM unpublished results].

The source of ROS is probably the same in ammonium- and Pi-starved cells because ammonium-starved cells also continue to consume glucose, but at a rate twice lower than in Pi-starved cells [56]. A simple interpretation of this low metabolic rate is that the PTS^Ntr^ system, which is mainly phosphorylated in ammonium-starved cells [76], competes with the glucose-specific phospho*enol*pyruvate phosphotransferase system (PTS^Glc^) for the common phosphoryl donor, phospho*enol*pyruvate (PEP) (Fig. 1). Slow transport of glucose could hamper fermentative overflow metabolism (Pta-AckA pathway) in favour of aerobic metabolism (TCA-ETC pathway), which could increase the production of ROS. Hydroxyl radical could indirectly activate SoxR, provided that the NADPH-dependent Rsx-reducing system protects SoxR [2Fe-2S] cluster from over-oxidation. However, reduced overflow metabolism (Pta-AckA pathway) could decrease AcP and Ace levels and thus prevent CpxR and RpoE activation. Moreover, the phosphorylation of PtsN could prevent induction of the LexA regulon. PtsN^Pi^ increases the activity of both Trk (K^+^:H^+^ symport) and CvrA (K^+^ efflux) [88] rather than that of KdpD, which could cause a net accumulation of protons inside the cell. A decrease in the pHi could increase the binding affinity of LexA to LexA box and prevent the expression of the LexA regulon. In sum, the phosphorylation of PtsN could directly (H^+^ accumulation) and indirectly (competition for PEP) account for the strong induction of the SoxRS regulon and the weak induction of the CpxR, RpoE and LexA regulons in ammonium-starved cells.

## Survival of glucose-starved cells

### Activation of the SoxR and OxyR regulons

When cells begin the transition to stationary phase because of a decrease in glucose levels in the medium, the PTS^Glc^ system triggers the accumulation of cyclic AMP (cAMP). Binding of cAMP to the cAMP-receptor protein (CRP) induces genes that help to scavenge and metabolize glucose through PTS^Glc^ (*pts*), PDH (*aceEF-lpd*) and TCA (*acnB* and *sdh*) [1, 89, 90]. Metabolism through the PDH-TCA-ETC pathway could transiently increase ROS levels [14] before metabolism switches towards Ace excretion [38]. The burst of hydrogen peroxide and hydroxyl radical could activate OxyR and SoxR, respectively. However, under glucose-limited conditions, PYR and fructose-1,6-bisphosphate/fructose-1-phosphate concentrations decrease rapidly, which activates PdhR and Cra [1, 89–91]. Cra without ligand can help SoxS to induce *poxB* − rather than *sodA* and *zwf* as in Pi-starved cells − whereas PdhR without ligand inhibits the synthesis of PDH [89, 92, 93]. Switching PYR flux from PDH to PoxB could rapidly stop the production of ROS [28]. The hypothesis of a weak production of ROS for a limited period of time is in good agreement with the finding that the viability of oxidative-stress sensitive mutants is barely (*ahpCF katE*) or weakly affected (*katG katE dps*) under glucose-starvation conditions. However, the viability of *oxyR* single mutants and *oxyR rpoS* double mutants decreases markedly at the entry into stationary phase, which suggests that induction of gene(s) of the OxyR regulon could help glucose-starved cells to resist to low levels of ROS during the transition to stationary phase [14].

### Low levels of ATP and iron: activation of the Fur regulon

Glucose-starved cells accumulate high levels of the sRNA RyhB, whereas RyhB is not detected in Pi-starved cells (Julia Bos and PLM unpublished results). The repressor Fur (*F*erric *u*ptake *r*egulator) controls the transcription of *ryhB* [94–96]. Therefore, RyhB accumulation indicates that glucose-starved cells behave as cells starved of iron in which Fur is inactivate (apo-Fur), whereas Fur is active (metalated) in Pi-starved cells [97]. Because glucose- and Pi-starved cells are incubated in a medium containing the same concentration of iron, a simple interpretation of these data is that glucose-starved cells cannot transport iron inside the cell.

The activity of iron transporters requires a PMF and ATP [4]. However, glucose-starved cells exhibit a weak energy-generating metabolism following the bypass of PDH by PoxB. PoxB is a non-energy conserving enzyme. Biogenesis of the alternative ETC composed of PoxB-Q-Cyo/Cbd generates a weak PMF [7]. Low PMF and low ATP levels in glucose-starved cells [98, 99] could therefore primarily account for low activity of iron transport systems, low levels of iron inside the cell and induction of the Fur regulon (e.g. *ryhB*, *fep* and *suf*).

RyhB helps maintain the viability of iron-starved cells both by stimulating the synthesis of enterobactin that scavenges iron, and by inhibiting the synthesis of most Fe-proteins but a few ‘essential’ FeS proteins − e.g. ribonucleotide reductase that permits dNTP synthesis [94, 100]. The synthesis of ‘essential’ FeS proteins could primarily require the activity of the Ent-Fep-Suf system. Fep helps transport Ent(Fe^3+^) complexes [4]. Suf can replace the FeS assembly-delivery Isc system normally used in growing cells [15, 80, 94]. A role for the Suf system under iron-deficient and oxidative-stress conditions is in good agreement with the fact that the *sufA-E* operon is negatively controlled by Fur and positively controlled by OxyR [80, 94]. It is therefore tempting to speculate that the viability of glucose-starved cells could depend on induction of the *sufA-E* operon, which could help to synthesize FeS proteins required to mitigate oxidative stress.

### Iron sulphur/SAM-dependent methyltransferases increase translational fidelity

Pi-starved cells degrade both threonine to S-adenosyl-l-methionine (SAM), and arginine to putrescine, thereby producing spermidine [81]. Spermidine, which is mostly associated with RNA, can scavenge hydroxyl radical (HO•), thereby protecting RNA from oxidative damage [101, 102]. Oxidation of spermidine gives rise to spermidine dialdehyde [102]. It is therefore likely that TBARS detected in Pi-starved cells (i.e. dialdehydes such as molondialdehyde) [10, 28] could be mainly spermidine dialdehyde bound to polyribosomes.

In sharp contrast, glucose-starved cells degrade threonine but not arginine (PLM unpublished results), which could improve SAM rather than spermidine synthesis. Glucose-starved do not accumulate TBARS [28]. If glucose-starved cells were accumulating SAM, the primary donor of the methyl group [103], abnormal alkylated bases in DNA (e.g. 7meG and 3meA) could be produced, which could induce the Ada adaptive response whose role is to eliminate such mutagenic DNA lesions [104]. In fact, glucose-starved cells are resistant to alkylating agents, which suggests that excess SAM triggers the Ada response in glucose-starved cells (PLM unpublished results).

Considering the accumulation of SAM and activation of the Suf system, it is tempting to speculate that the synthesis of SAM-dependent iron-sulphur enzymes (i.e. radical SAM methyltransferases) could play a key role in the viability of glucose-starved cells. Radical SAM methyltransferases such as RlmN and MiaB methylate tRNAs, which enhances translational fidelity and thus prevents the synthesis of abnormal proteins [49, 103, 105]. It has been suggested that death of glucose-starved cells could primarily result from the accumulation of abnormal proteins, as a result of a shortage of charged tRNAs, rather than from an increase in the production of ROS [106]. Aberrant and misfolded proteins could be prone to oxidative damage, which could account for the accumulation of protein carbonyl derivatives (i.e. oxidative fragmentation products) in glucose-starved cells [12, 106].

An increase in translational fidelity could therefore prevent the synthesis of misfolded proteins sensitive to oxidative damage. The pre-emptive role of radical SAM methyltransferases could be critical if glucose-starved cells would contain low ATP levels, which could prevent the activity of ClpP and Lon ATP-dependent proteases that normally degrade misfolded proteins [53].

## Conclusion

In nature, *E. coli* can be starved of Pi in the small intestine [45, 56] and, following faecal deposition, of ammonium and glucose in soils and freshwater, respectively [107]. *E. coli* evolve different strategies to survive during the transition from the exponential growth phase to the stationary phase. Pi- and ammonium-starved cells, which can metabolize high levels of glucose, should fight against high levels of ROS. Pi-starved cells, which produce the unmodified form of PtsN, use several stress regulons (LexA, Cpx and RpoE) in addition to SoxRS and OxyR oxidative stress regulons to mitigate oxidative damage. Ammonium-starved cells, which produce the phosphorylated form of PtsN, rely primarily on the SoxRS-Rsx system to mitigate oxidative damage. Glucose-starved cells could use a new strategy based primarily on the production of SAM/FeS methyltransferases that could help to prevent the synthesis of abnormal proteins prone to oxidation.

## References

[1] Kochanowski K, Gerosa L, Brunner SF, Christodoulou D, Nikolaev YV (2017). Few regulatory metabolites coordinate expression of central metabolic genes in *Escherichia coli*. Mol Syst Biol.

[2] Lamarche MG, Wanner BL, Crépin S, Harel J (2008). The phosphate regulon and bacterial virulence: a regulatory network connecting phosphate homeostasis and pathogenesis. FEMS Microbiol Rev.

[3] Altendorf K, Booth IR, Gralla J, Greie J-C, Rosenthal AZ (2009). Osmotic Stress. EcoSal Plus.

[4] Nies DH, Grass G (2009). Transition metal homeostasis. EcoSal Plus.

[5] Krulwich TA, Sachs G, Padan E (2011). Molecular aspects of bacterial pH sensing and homeostasis. Nat Rev Microbiol.

[6] Ruiz N, Silhavy TJ (2005). Sensing external stress: watchdogs of the *Escherichia coli* cell envelope. Curr Opin Microbiol.

[7] Unden G, Steinmetz PA, Degreif-Dünnwald P (2014). The aerobic and anaerobic respiratory chain of *Escherichia coli* and *Salmonella enterica*: enzymes and energetics. EcoSal Plus.

[8] Moreau P, Bailone A, Devoret R (1976). Prophage lambda induction of *Escherichia coli* K12 envA uvrB: a highly sensitive test for potential carcinogens. Proc Natl Acad Sci.

[9] Baharoglu Z, Mazel D (2014). SOS, the formidable strategy of bacteria against aggressions. FEMS Microbiol Rev.

[10] Imlay JA (2019). Where in the world do bacteria experience oxidative stress?. Environ Microbiol.

[11] Ketcham A, Freddolino PL, Tavazoie S (2022). Intracellular acidification is a hallmark of thymineless death in *E. coli*. PLoS Genet.

[12] Berlett BS, Stadtman ER (1997). Protein oxidation in aging, disease, and oxidative stress. J Biol Chem.

[13] Cadet J, Davies KJA, Medeiros MHG, Di Mascio P, Wagner JR (2017). Formation and repair of oxidatively generated damage in cellular DNA. Free Radic Biol Med.

[14] Moreau PL, Gérard F, Lutz NW, Cozzone P (2001). Non-growing *Escherichia coli* cells starved for glucose or phosphate use different mechanisms to survive oxidative stress. Mol Microbiol.

[15] Imlay JA, Slauch JM (2009). Oxidative stress. EcoSal Plus.

[16] Weber H, Polen T, Heuveling J, Wendisch VF, Hengge R (2005). Genome-wide analysis of the general stress response network in *Escherichia coli*: sigmaS-dependent genes, promoters, and sigma factor selectivity. J Bacteriol.

[17] Korshunov S, Imlay JA (2006). Detection and quantification of superoxide formed within the periplasm of *Escherichia coli*. J Bacteriol.

[18] Battesti A, Majdalani N, Gottesman S (2011). The RpoS-mediated general stress response in *Escherichia coli*. Annu Rev Microbiol.

[19] Gottesman S (2019). Trouble is coming: signaling pathways that regulate general stress responses in bacteria. J Biol Chem.

[20] Wolfe AJ, Parikh N, Lima BP, Zemaitaitis B (2008). Signal integration by the two-component signal transduction response regulator CpxR. J Bacteriol.

[21] Lima BP, Thanh Huyen TT, Bäsell K, Becher D, Antelmann H (2012). Inhibition of acetyl phosphate-dependent transcription by an acetylatable lysine on RNA polymerase. J Biol Chem.

[22] Gopalkrishnan S, Nicoloff H, Ades SE (2014). Co-ordinated regulation of the extracytoplasmic stress factor, sigmaE, with other *Escherichia coli* sigma factors by (p)ppGpp and DksA may be achieved by specific regulation of individual holoenzymes. Mol Microbiol.

[23] Sineva E, Savkina M, Ades SE (2017). Themes and variations in gene regulation by extracytoplasmic function (ECF) sigma factors. Current Opin Microbiol.

[24] Dri A-M, Moreau PL (1993). Phosphate starvation and low temperature as well as ultraviolet irradiation transcriptionally induce the *Escherichia coli* LexA-controlled gene sfiA. Mol Microbiol.

[25] Moreau PL (2014). Protective role of the RpoE (σE) and Cpx envelope stress responses against gentamicin killing of nongrowing *Escherichia coli* incubated under aerobic, phosphate starvation conditions. FEMS Microbiol Lett.

[26] Moreau PL, Loiseau L (2016). Characterization of acetic acid-detoxifying *Escherichia coli* evolved under phosphate starvation conditions. Microb Cell Fact.

[27] Imlay JA (2015). Transcription factors that defend bacteria against reactive oxygen species. Annu Rev Microbiol.

[28] Moreau PL (2004). Diversion of the metabolic flux from pyruvate dehydrogenase to pyruvate oxidase decreases oxidative stress during glucose metabolism in nongrowing *Escherichia coli* cells incubated under aerobic, phosphate starvation conditions. J Bacteriol.

[29] Osterman A (2009). Biogenesis and homeostasis of nicotinamide adenine dinucleotide cofactor. EcoSal Plus.

[30] Romeo T, Snoep JL (2005). Glycolysis and flux control. EcoSal Plus.

[31] Cronan Jr JE, Laporte D, Stewart V (2005). Tricarboxylic acid cycle and glyoxylate bypass. EcoSal Plus.

[32] Korshunov S, Imlay JA (2010). Two sources of endogenous hydrogen peroxide in *Escherichia coli*. Mol Microbiol.

[33] Park S, You X, Imlay JA (2005). Substantial DNA damage from submicromolar intracellular hydrogen peroxide detected in Hpx- mutants of *Escherichia coli*. Proc Natl Acad Sci.

[34] Sontz PA, Mui TP, Fuss JO, Tainer JA, Barton JK (2012). DNA charge transport as a first step in coordinating the detection of lesions by repair proteins. Proc Natl Acad Sci.

[35] May KL, Lehman KM, Mitchell AM, Grabowicz M (2019). A stress response monitoring lipoprotein trafficking to the outer membrane. mBio.

[36] Guest RL, Wang J, Wong JL, Raivio TL (2017). A bacterial stress response regulates respiratory protein complexes to control envelope stress adaptation. J Bacteriol.

[37] Mulkidjanian AY, Heberle J, Cherepanov DA (2006). Protons @ interfaces: implications for biological energy conversion. Biochimica et Biophysica Acta.

[38] Wolfe AJ (2005). The acetate switch. Microbiol Mol Biol Rev.

[39] Dri A-M, Moreau PL (1994). Control of the LexA regulon by pH: evidence for a reversible inactivation of the LexA repressor during the growth cycle of *Escherichia coli*. Mol Microbiol.

[40] Haverkorn van Rijsewijk BRB, Nanchen A, Nallet S, Kleijn RJ, Sauer U (2011). Large-scale 13C-flux analysis reveals distinct transcriptional control of respiratory and fermentative metabolism in *Escherichia coli*. Mol Syst Biol.

[41] Basan M, Hui S, Okano H, Zhang Z, Shen Y (2015). Overflow metabolism in *Escherichia coli* results from efficient proteome allocation. Nature.

[42] Cariss SJL, Tayler AE, Avison MB (2008). Defining the growth conditions and promoter-proximal DNA sequences required for activation of gene expression by CreBC in *Escherichia coli*. J Bacteriol.

[43] McCleary WR, Stock JB (1994). Acetyl phosphate and the activation of two-component response regulators. J Biol Chem.

[44] Vuppada RK, Hansen CR, Strickland KAP, Kelly KM, McCleary WR (2018). Phosphate signaling through alternate conformations of the PstSCAB phosphate transporter. BMC Microbiol.

[45] Guillemet ML, Moreau PL (2012). Activation of the cryptic PhnE permease promotes rapid adaptive evolution in a population of *Escherichia coli* K-12 starved for phosphate. J Bacteriol.

[46] Germain E, Guiraud P, Byrne D, Douzi B, Djendli M (2019). YtfK activates the stringent response by triggering the alarmone synthetase SpoT in *Escherichia coli*. Nat Commun.

[47] Fernández-Coll L, Cashel M (2018). Contributions of SpoT hydrolase, SpoT synthetase, and RelA synthetase to carbon source diauxic growth transitions in *Escherichia coli*. Front Microbiol.

[48] Wu C, Balakrishnan R, Braniff N, Mori M, Manzanarez G (2022). Cellular perception of growth rate and the mechanistic origin of bacterial growth law. Proc Natl Acad Sci.

[49] Sanchez-Vazquez P, Dewey CN, Kitten N, Ross W, Gourse RL (2019). Genome-wide effects on *Escherichia coli* transcription from ppGpp binding to its two sites on RNA polymerase. Proc Natl Acad Sci.

[50] Leblanc SKD, Oates CW, Raivio TL (2011). Characterization of the induction and cellular role of the BaeSR two-component envelope stress response of *Escherichia coli*. J Bacteriol.

[51] Choudhary KS, Kleinmanns JA, Decker K, Sastry AV, Gao Y (2020). Elucidation of regulatory modes for five two-component systems in *Escherichia coli* reveals novel relationships. mSystems.

[52] Rhodius VA, Suh WC, Nonaka G, West J, Gross CA (2006). Conserved and variable functions of the sigmaE stress response in related genomes. PLoS Biol.

[53] Gur E, Biran D, Ron EZ (2011). Regulated proteolysis in Gram-negative bacteria--how and when?. Nat Rev Microbiol.

[54] Yakhnin H, Aichele R, Ades SE, Romeo T, Babitzke P (2017). Circuitry linking the global Csr- and σ^E^-dependent cell envelope stress response systems. J Bacteriol.

[55] Camacho MI, Alvarez AF, Chavez RG, Romeo T, Merino E (2015). Effects of the global regulator CsrA on the BarA/UvrY two-component signaling system. J Bacteriol.

[56] Moreau PL (2007). The lysine decarboxylase CadA protects *Escherichia coli* starved of phosphate against fermentation acids. J Bacteriol.

[57] Sassanfar M, Roberts JW (1990). Nature of the SOS-inducing signal in *Escherichia coli*. The involvement of DNA replication. J Mol Biol.

[58] Moreau PL (1987). Effects of overproduction of single-stranded DNA-binding protein on RecA protein-dependent processes in *Escherichia coli*. J Mol Biol.

[59] Moreau PL, Carlier M-F (1989). RecA protein-promoted cleavage of LexA repressor in the presence of ADP and structural analogues of inorganic phosphate, the fluoride complexes of aluminum and beryllium. J Biol Chem.

[60] Umezu K, Kolodner RD (1994). Protein interactions in genetic recombination in *Escherichia coli*. Interactions involving RecO and RecR overcome the inhibition of RecA by single-stranded DNA-binding protein. J Biol Chem.

[61] Bell JC, Liu B, Kowalczykowski SC (2015). Imaging and energetics of single SSB-ssDNA molecules reveal intramolecular condensation and insight into RecOR function. Elife.

[62] Godoy VG, Jarosz DF, Simon SM, Abyzov A, Ilyin V (2007). UmuD and RecA directly modulate the mutagenic potential of the Y family DNA polymerase DinB. Mol Cell.

[63] Joseph AM, Badrinarayanan A (2020). Visualizing mutagenic repair: novel insights into bacterial translesion synthesis. FEMS Microbiol Rev.

[64] Tuan PM, Gilhooly NS, Marians KJ, Kowalczykowski SC (2022). Direct visualization of translesion DNA synthesis polymerase IV at the replisome. Proc Natl Acad Sci.

[65] Moreau PL (1988). Overproduction of single-stranded-DNA-binding protein specifically inhibits recombination of UV-irradiated bacteriophage DNA in *Escherichia coli*. J Bacteriol.

[66] Dri A-M, Moreau PL (1991). Properties of RecA441 protein reveal a possible role for RecF and SSB proteins in *Escherichia coli*. Mol Gen Genet.

[67] Henrikus SS, Henry C, Ghodke H, Wood EA, Mbele N (2019). RecFOR epistasis group: RecF and RecO have distinct localizations and functions in *Escherichia coli*. Nucleic Acids Res.

[68] Lin L-L, Little JW (1988). Isolation and characterization of noncleavable (ind-) mutants of the lexa repressor of *Escherichia coli* K-12. J Bacteriol.

[69] Luo Y, Pfuetzner RA, Mosimann S, Paetzel M, Frey EA (2001). Crystal structure of lexa: a conformational switch for regulation of self-cleavage. Cell.

[70] Jones EC, Uphoff S (2021). Single-molecule imaging of LexA degradation in *Escherichia coli* elucidates regulatory mechanisms and heterogeneity of the SOS response. Nat Microbiol.

[71] Zhang APP, Pigli YZ, Rice PA (2010). Structure of the LexA-DNA complex and implications for SOS box measurement. Nature.

[72] Winkelmann I, Uzdavinys P, Kenney IM, Brock J, Meier PF (2022). Crystal structure of the Na+/H+ antiporter NhaA at active pH reveals the mechanistic basis for pH sensing. Nat Commun.

[73] Booth JA, Špírek M, Lobie TA, Skarstad K, Krejci L (2020). Antibiotic-induced DNA damage results in a controlled loss of pH homeostasis and genome instability. Sci Rep.

[74] Schramke H, Laermann V, Tegetmeyer HE, Brachmann A, Jung K (2017). Revisiting regulation of potassium homeostasis in *Escherichia coli*: the connection to phosphate limitation. MicrobiologyOpen.

[75] Lüttmann D, Göpel Y, Görke B (2012). The phosphotransferase protein EIIA(Ntr) modulates the phosphate starvation response through interaction with histidine kinase PhoR in *Escherichia coli*. Mol Microbiol.

[76] Lee C-R, Park Y-H, Kim M, Kim Y-R, Park S (2013). Reciprocal regulation of the autophosphorylation of enzyme INtr by glutamine and α-ketoglutarate in *Escherichia coli*. Mol Microbiol.

[77] Euro L, Belevich G, Wikström M, Verkhovskaya M (2009). High affinity cation-binding sites in Complex I from *Escherichia coli*. Biochim Biophys Acta.

[78] Sun Z, Cagliero C, Izard J, Chen Y, Zhou YN (2019). Density of σ70 promoter-like sites in the intergenic regions dictates the redistribution of RNA polymerase during osmotic stress in *Escherichia coli*. Nucleic Acids Res.

[79] Anand A, Olson CA, Sastry AV, Patel A, Szubin R (2021). Restoration of fitness lost due to dysregulation of the pyruvate dehydrogenase complex is triggered by ribosomal binding site modifications. Cell Rep.

[80] Py B, Moreau PL, Barras F (2011). Fe–S clusters, fragile sentinels of the cell. Current Opin Microbiol.

[81] Gérard F, Dri A-M, Moreau PL (1999). Role of *Escherichia coli* RpoS, LexA and H-NS global regulators in metabolism and survival under aerobic, phosphate-starvation conditions. Microbiology.

[82] Anand A, Chen K, Yang L, Sastry AV, Olson CA (2019). Adaptive evolution reveals a tradeoff between growth rate and oxidative stress during naphthoquinone-based aerobic respiration. Proc Natl Acad Sci.

[83] Pin C, Rolfe MD, Muñoz-Cuevas M, Hinton JCD, Peck MW (2009). Network analysis of the transcriptional pattern of young and old cells of *Escherichia coli* during lag phase. BMC Syst Biol.

[84] Fritz G, Walker N, Gerland U (2019). Heterogeneous timing of gene induction as a regulation strategy. J Mol Biol.

[85] Zamora M, Ziegler CA, Freddolino PL, Wolfe AJ (2020). A thermosensitive, phase-variable epigenetic switch: pap revisited. Microbiol Mol Biol Rev.

[86] Mitchell A, Romano GH, Groisman B, Yona A, Dekel E (2009). Adaptive prediction of environmental changes by microorganisms. Nature.

[87] Vemparala B, Valiya Parambathu A, Saini DK, Dixit NM (2022). An evolutionary paradigm favoring cross talk between bacterial two-component signaling systems. mSystems.

[88] Sharma R, Shimada T, Mishra VK, Upreti S, Sardesai AA (2016). Growth inhibition by external potassium of *Escherichia coli* lacking PtsN (EIIANtr) is caused by potassium limitation mediated by YcgO. J Bacteriol.

[89] Kotte O, Zaugg JB, Heinemann M (2010). Bacterial adaptation through distributed sensing of metabolic fluxes. Mol Syst Biol.

[90] Kim D, Seo SW, Gao Y, Nam H, Guzman GI (2018). Systems assessment of transcriptional regulation on central carbon metabolism by Cra and CRP. Nucleic Acids Res.

[91] Bley Folly B, Ortega AD, Hubmann G, Bonsing-Vedelaar S, Wijma HJ (2018). Assessment of the interaction between the flux-signaling metabolite fructose-1,6-bisphosphate and the bacterial transcription factors CggR and Cra. Mol Microbiol.

[92] Shimada T, Yamamoto K, Ishihama A (2011). Novel members of the Cra regulon involved in carbon metabolism in *Escherichia coli*. J Bacteriol.

[93] Son Y-J, Phue J-N, Trinh LB, Lee SJ, Shiloach J (2011). The role of Cra in regulating acetate excretion and osmotic tolerance in *E. coli* K-12 and *E. coli* B at high density growth. Microb Cell Fact.

[94] Massé E, Vanderpool CK, Gottesman S (2005). Effect of RyhB small RNA on global iron use in *Escherichia coli*. J Bacteriol.

[95] Seo SW, Kim D, Latif H, O’Brien EJ, Szubin R (2014). Deciphering Fur transcriptional regulatory network highlights its complex role beyond iron metabolism in *Escherichia coli*. Nat Commun.

[96] Fontenot CR, Tasnim H, Valdes KA, Popescu CV, Ding H (2020). Ferric uptake regulator (Fur) reversibly binds a [2Fe-2S] cluster to sense intracellular iron homeostasis in *Escherichia coli*. J Biol Chem.

[97] Guillemet ML, Moreau PL (2008). Fur-dependent detoxification of organic acids by rpoS mutants during prolonged incubation under aerobic, phosphate starvation conditions. J Bacteriol.

[98] Manuse S, Shan Y, Canas-Duarte SJ, Bakshi S, Sun W-S (2021). Bacterial persisters are a stochastically formed subpopulation of low-energy cells. PLoS Biol.

[99] Peterson CN, Levchenko I, Rabinowitz JD, Baker TA, Silhavy TJ (2012). RpoS proteolysis is controlled directly by ATP levels in *Escherichia coli*. Genes Dev.

[100] Salvail H, Lanthier-Bourbonnais P, Sobota JM, Caza M, Benjamin J-A (2010). A small RNA promotes siderophore production through transcriptional and metabolic remodeling. Proc Natl Acad Sci.

[101] Winther KS, Sørensen MA, Svenningsen SL (2021). Polyamines are required for tRNA anticodon modification in *Escherichia coli*. J Mol Biol.

[102] Ha HC, Sirisoma NS, Kuppusamy P, Zweier JL, Woster PM (1998). The natural polyamine spermine functions directly as a free radical scavenger. Proc Natl Acad Sci.

[103] Bauerle MR, Schwalm EL, Booker S (2015). Mechanistic diversity of radical S-adenosylmethionine (SAM)-dependent methylation. J Biol Chem.

[104] Mielecki D, Grzesiuk E (2014). Ada response - a strategy for repair of alkylated DNA in bacteria. FEMS Microbiol Lett.

[105] Esakova OA, Grove TL, Yennawar NH, Arcinas AJ, Wang B (2021). Structural basis for tRNA methylthiolation by the radical SAM enzyme MiaB. Nature.

[106] Ballesteros M, Fredriksson A, Henriksson J, Nyström T (2001). Bacterial senescence: protein oxidation in non-proliferating cells is dictated by the accuracy of the ribosomes. EMBO J.

[107] Bergholz PW, Noar JD, Buckley DH (2011). Environmental patterns are imposed on the population structure of *Escherichia coli* after fecal deposition. Appl Environ Microbiol.

